# Associations of Adjunctive Multistrain Probiotic/Prebiotic Supplementation with Microbiological Eradication and Semen Parameters in Infertile Men with Male Tract Infections: A Retrospective Cohort Study

**DOI:** 10.3390/life16101683

**Published:** 2026-10-09

**Authors:** Giuseppe Grande, Andrea Graziani, Rossella Mazzilli, Davide Abate, Luca De Toni, Alberto Ferlin

**Affiliations:** 1 Unit of Andrology and Reproductive Medicine, Department of Systems Medicine, University Hospital of Padova, 35128 Padova, Italy; andrea.graziani.1@unipd.it (A.G.);; 2 Department of Medicine, University of Padova, 35128 Padova, Italy; luca.detoni@unipd.it; 3 Department of Experimental Medicine, Sapienza University of Rome, 00161 Rome, Italy; rossella.mazzilli@uniroma1.it; 4 Department of Molecular Medicine, University of Padova, 35128 Padova, Italy

**Keywords:** male factor infertility, semen infections, antibiotic therapy, microbiota, probiotic, prebiotic

## Abstract

Background: Male tract infections (MTIs) represent a relevant cause of male factor infertility, yet microbiological persistence after antibiotic therapy remains common. This retrospective observational cohort study evaluated whether adjunctive multistrain probiotic/prebiotic supplementation was associated with microbiological and seminal outcomes in infertile men with microbiologically confirmed MTIs. Methods: One hundred infertile men with confirmed MTIs received either antibiotic therapy alone (n = 50) or antibiotics plus sequential multistrain probiotic/prebiotic supplementation (n = 50). The primary outcome was microbiological eradication. Secondary outcomes included changes in sperm concentration and total sperm count. Multivariable analyses adjusted for infertility duration, female partner age, pathogen category, and baseline semen parameters. Results: Microbiological eradication was achieved in 92.0% of patients receiving combined therapy versus 56.0% receiving antibiotics alone (adjusted OR 7.29, 95% CI 2.19–24.23; *p* = 0.001). Combined therapy was associated with greater improvements in sperm concentration and total sperm count, both when defined as any increase and as a ≥50% increase from baseline (all *p* < 0.01). These findings remained consistent across continuous analyses, pathogen-specific subgroup analyses, and sensitivity analyses excluding mixed infections. Conclusions: Adjunctive multistrain probiotic/prebiotic supplementation was associated with higher microbiological eradication rates and greater improvements in semen quality than antibiotic therapy alone. Prospective randomized controlled trials are warranted to confirm these findings and define the role of microbiota-targeted therapies in managing infertility associated with MTIs.

## 1. Introduction

Couple infertility is defined as the failure to achieve conception after at least 12 months of regular unprotected intercourse [1,2,3,4,5]. Representing a major public health concern, it affects millions of couples and carries clinical, psychological, social, and economic implications. The inability to conceive may result from female, male, or combined factors [6]. Among these, male factor infertility (MFI) constitutes a major contributor, affecting about half of the infertile couples and representing a considerable challenge in reproductive medicine [2,7,8,9,10].

The etiology of MFI is multifactorial and includes genetic defects, endocrine disorders, infection/inflammation disease, lifestyle-related influences, varicocele, and several other pathological conditions [4,8,9,11,12,13]. Among the recognized causes of MFI, male tract inflammations/infections play a particularly important role and may account for up to 20% of infertility cases in men [7,14]. Owing to their relatively high prevalence and potential impact on reproductive function, the identification and management of male tract infections (MTIs) should represent an integral component of the diagnostic work-up in patients presenting with MFI [14].

A major challenge in the clinical management of MTIs is their frequently silent presentation. Indeed, these infections often result in delayed diagnosis and inadequate treatment. While some patients may report signs or symptoms of infection, many infected individuals remain completely asymptomatic. Numerous pathogens, including bacteria, viruses, fungi, and human papillomavirus (HPV), have been implicated in infections of the male reproductive tract and may adversely affect fertility [8,15,16].

The largely asymptomatic nature of MTIs makes their detection particularly difficult without a comprehensive clinical assessment [14]. Furthermore, reproductive outcomes may vary considerably depending on several factors, including the causative microorganism, the duration and progression of the infection, and the specific anatomical site involved within the male reproductive system. Several biological pathways have been proposed to explain the detrimental effects of MTIs on male reproductive potential. Collectively, these alterations can significantly reduce male reproductive capacity and contribute to the development of MFI [17].

According to the guidelines of the European Association of Urology, antibiotic administration remains the primary therapeutic approach for bacterial MTIs [18]. Nevertheless, treatment selection should be individualized, taking into account the specific pathogen involved, patient-related factors such as renal impairment or drug hypersensitivity, and the results of antimicrobial susceptibility testing to maximize treatment efficacy.

However, antibiotic treatment does not invariably lead to complete pathogen eradication. Evidence suggests that persistent infection remains detectable in semen cultures following therapy in approximately 40–50% of treated individuals, indicating a substantial rate of therapeutic failure [19,20]. In light of these limitations, increasing attention has been directed toward adjunctive therapeutic approaches capable of enhancing infection clearance. One emerging area of interest concerns the use of probiotics as a complementary strategy to conventional antibiotic treatment [17,20], since gut dysbiosis has frequently been reported in patients with recurrent MTIs, as previously documented [21], thus supporting the hypothesis that modulation of the intestinal microbiota may influence the management of chronic infections affecting the male reproductive tract.

In fact, several microorganisms, including bacteria, yeasts, and viruses, coexist within the human body and across different organ systems, while the collective human microbiota contributes more than 150 times as much genetic information [22,23]. In addition, In addition, site-specific variations exist in microbiota composition, with the gut microbiota playing a particularly important role in maintaining human health [24,25]. Indeed, the gut microbiota and its products may modulate and affect multiple organs other than the gut, including the male reproductive system [17,26,27,28,29,30,31,32,33,34,35].

The aim of the present study was therefore to evaluate whether adjunctive sequential multistrain probiotic/prebiotic supplementation was associated with microbiological eradication and improved semen parameters in infertile men receiving antibiotic therapy for clinically relevant MTIs.

## 2. Materials and Methods

The study was approved by the Institutional Review Board and conducted in accordance with the guidelines of the Declaration of Helsinki. Informed consent was obtained from each patient.

### 2.1. Patients

We retrospectively evaluated in the study a population of 20- to 55-year-old patients seeking medical care for primary couple infertility. All patients underwent familial and personal history, physical examination, semen analysis according to the WHO manual for semen examination [1], fasting morning hormonal evaluation (total testosterone—TT, follicle-stimulating hormone—FSH, luteinizing hormone—LH [20]), testicular ultrasound (US) to measure testicular volumes, echogenicity, homogeneity, and vascularization, characteristic of the epididymis and the proximal tract of vas deferens, and the presence of varicocele [21,22]. Semen microbiological evaluation (for general bacteria and atypical bacteria) was performed. Each specimen was cultured in 3 sets of agar plates—Columbia agar 5% sheep blood, chocolate agar and Sabouraud dextrose agar (Becton Dickinson, Franklin Lakes, USA)- for 48 h at 37 °C. Specimens were seeded in agar plates using an automated seeding device (WASPlab—Copan, Brescia, Italy) providing a semiquantitative range of microbial growth. Significant microbial growth (>10^4^ colony forming units (CFUs) were isolated and identified using MALDI-TOF (Bruker, Billerica, USA) and tested for antimicrobial susceptibility (providing the minimum inhibitory concentration, MIC, Microscan walkaway, Beckman Coulter, Brea, USA). *Mycoplasma* spp. *Neisseria gonorrhoeae*, *Trichomonas vaginalis* and *Chlamydia trachomatis* were identified using an automated molecular testing BD MAX, Becton Dickinson, providing a quantitative result as positive, negative or unresolved. Typical pathogens included common enteric and urogenital bacteria, including members of the Enterobacterales order, *Enterococcus* spp., and *Streptococcus* spp. Atypical pathogens included *Ureaplasma* spp., *Mycoplasma* spp., and *Chlamydia trachomatis*. Mixed infections were defined by the concomitant detection of at least one typical and one atypical pathogen.

To distinguish clinically relevant MTIs from isolated semen microbiological positivity, eligibility required both microbiological evidence of infection together with additional clinical, seminal, and/or ultrasonographic evidence compatible with infection/inflammation of the male reproductive tract, as previously reported [14]. We therefore enrolled for this study infertile male patients with normal testicular volumes, normal (FSH values (<8 mUI/L), a positive semen microbiological assessment for at least one typical and/or atypical pathogen, and at least one of the following: (i) anamnesis and/or physical examination suggestive for infection/inflammation/obstruction and/or (ii) semen signs of suspected infection/inflammation (semen volume < 1.4 or >6.2 mL, semen pH < 7.2 or >8.5, increased semen viscosity and/or altered semen fluidification and/or presence of leukocytospermia > 106/mL and/or presence of sperm agglutination and/or presence of ASAs) and/or (iii) epididymal US features of inflammation/obstruction (>2 of the following US signs: increase in size of the head of epididymis (cranio-caudal diameter > 12 mm) and/or of the body (>5 mm) and/or of the tail (>6 mm) (mono or bilateral); presence of multiple microcysts in the head and/or tail (mono or bilateral); low or high echogenicity (mono or bilateral); large mono or bilateral hydrocele; enlargement in superior part of the cephalic tract and superior/inferior part ratio > 1; unchanged anteroposterior diameter of tail after ejaculation) [14,36,37]. This integrated approach was intended to minimize misclassification of incidental microbial detection or colonization as clinically relevant MTI and corresponds to the recently proposed infection/inflammation-related MFI phenotype (Category I) [14].

Exclusion criteria included US-based mean testicular volume < 11.5 mL, FSH > 8 mUI/L, fever, or drug use within 3 months prior to the enrollment in this study, low T concentrations in presence of low/inappropriately low gonadotropin levels and non-obstructive azoospermia. In addition, patients with US-evaluated high grade (above 3rd) varicocele were excluded from the current study, in order to avoid possible additional confounding factors [14].

### 2.2. Design of the Study

Patients were retrospectively identified among men evaluated at our Unit between October 2023 and April 2026. Patients receiving combined therapy and those receiving antibiotic therapy alone were recruited during the same study period and underwent the same diagnostic work-up, treatment selection procedure, and follow-up schedule. All consecutive patients fulfilling the predefined eligibility criteria during the study period were considered.

A total of 100 patients fulfilling the inclusion criteria have been considered for this study. Fifty patients received antibiotic treatment alone (controls), whereas 50 received the combined therapy (cases). Combined therapy was offered to all patients and the treatment option (probiotics and prebiotics + antibiotic or antibiotic alone) was chosen according to patient preference. The choice of adjunctive supplementation was not randomized and was based on patient preference after standardized counselling.

In order to reduce/avoid heterogeneity, the present study was restricted to patients whose identified pathogen(s) showed susceptibility to doxycycline and who consequently received the same doxycycline regimen (100 mg twice on day 1, followed by 100 mg once daily for the subsequent 8 days). Antimicrobial susceptibility was verified before treatment initiation. Patients requiring an alternative antibiotic because of antimicrobial resistance, contraindications, or other clinical reasons (n = 20) were excluded from the analytic cohort.

Doxycycline represents, in fact, the first-line treatment for atypical pathogens and the second-line treatment for typical pathogens. Furthermore, current evidence supports the increasing bacterial resistance to fluoroquinolones, which represent a concern in this field, as reported by current guidelines [18], so that doxycycline is often used in clinical practice both for typical and atypical bacteria. Thus, antibiotic type and duration were identical in the two treatment groups, irrespective of whether the infection was classified as typical or atypical.

Only patients with verified compliance and adherence were enrolled. Adverse events were retrospectively assessed from clinical records and follow-up documentation; no standardized prospective adverse-event collection instrument was used.

Fifty patients received a probiotic/prebiotic supplementation consisting of a commercial combination of probiotics (OMNi-BiOTiC^®^ 10 AAD: *Lactobacillus acidophilus* W55, *Lactobacillus acidophilus* W37, *Lactobacillus paracasei* W20, *Lactobacillus rhamnosus* W71, *Enterococcus faecium* W54, *Lactobacillus salivarius* W24, *Lactobacillus plantarum* W62, *Bifidobacterium bifidum* W23, *Lactobacillus plantarum* W1, *Bifidobacterium lactis* W51; Institut AllergoSan, Graz, Austria; 2 sachets daily for 10 days), during antibiotic treatment, followed by treatment with Lactobacilli (OMNi-BiOTiC^®^ FLORA plus+: *Lactobacillus crispatus* LBV88, *Lactobacillus rhamnosus* LBV96, *Lactobacillus gasseri*, *Lactobacillus jensenii* LBV116; Institut AllergoSan, Graz, Austria; 2 sachets daily for 30 days) and prebiotics (OMNi-LOGiC^®^ PLUS: Fructooligosaccharides (FOS) 5.26 g—Galactooligosaccharides (GOS) 3.79 g—Glucomannan; 3.00 g daily for 30 days; Institut AllergoSan, Graz, Austria). This regimen has been chosen with the aim to reduce the treatment time and to cover both the period of treatment with antibiotics and the following period, with the rationale of supporting microbiota recovery during and after antibiotic exposure.

After the treatment (one month following the end of the therapy—combined therapy for cases and antibiotic therapy for controls, 30 ± 5 days), a complete microbiological analysis (including culture for bacteria, mycoplasms, and chlamydia in semen was repeated. We moreover evaluated semen analysis three months following the end of the all therapy (90 ± 10 days—combined therapy for cases and antibiotic therapy for controls).

The primary outcome was microbiological eradication, defined as a negative post-treatment semen culture. Microbiological eradication was defined a priori as absence of the baseline-detected pathogen at the single prespecified post-treatment microbiological assessment. Repeated negative cultures were not required. Accordingly, the study assessed short-term microbiological eradication/persistence and was not designed to evaluate subsequent recurrence or reinfection.

All semen evaluations were performed in the same laboratory and evaluated by trained technicians and biologists [23]. Semen evaluations were performed according to WHO recommendations [1], with semen collection in the same laboratory and 2–5 days of sexual abstinence, under the same laboratory conditions. The baseline semen analysis used for the study was the last assessment performed before treatment initiation, within 30 days before the first antibiotic dose. Sperm concentration, total sperm count, total motility, progressive motility, and total motile sperm count (TMSC) were considered for the present analyses. TMSC was calculated as semen volume × sperm concentration × total motility/100.

### 2.3. Statistical Analysis

Statistical analyses were performed using R software, version 4.6.0 (R Foundation for Statistical Computing, Vienna, Austria). No formal a priori sample-size calculation was performed because this retrospective study included all eligible patients identified during the predefined study period. Continuous variables were evaluated for distributional characteristics and are reported as mean ± standard deviation or median (interquartile range), as appropriate. Age and female partner age were compared between groups using Welch’s *t*-test, whereas skewed continuous variables were compared using the Mann–Whitney U test. Categorical variables were summarized as number and percentage and compared using the chi-square test or Fisher’s exact test, as appropriate. The primary outcome was microbiological eradication, defined as a negative post-treatment semen culture. Secondary outcomes included any post-treatment increase and an increase of at least 50% over baseline in sperm concentration and total sperm count. Associations were expressed as odds ratios with 95% confidence intervals. Exploratory analyses additionally evaluated total and progressive sperm motility and total motile sperm count. Within-group pre- to post-treatment changes in semen parameters were assessed using the paired Wilcoxon signed-rank test, whereas between-group differences in absolute changes were evaluated using the Mann–Whitney U test. Multivariable logistic regression models were adjusted for infertility duration, female partner age, and pathogen category; models of seminal outcomes were additionally adjusted for the corresponding baseline semen parameter. Absolute changes in semen parameters were compared using the Mann–Whitney U test, with Cliff’s delta used as a non-parametric effect-size measure. Baseline-adjusted continuous-outcome analyses were performed using linear regression of log-transformed post-treatment values (log (1 + value)), adjusting for the corresponding log-transformed baseline value and the prespecified covariates, with HC3 heteroscedasticity-robust standard errors. Subgroup analyses were conducted according to typical or atypical pathogen category, including a treatment-by-pathogen interaction term. A sensitivity analysis excluded patients with mixed infections. To further address potential confounding related to the non-randomized treatment allocation, a post hoc propensity-score sensitivity analysis was performed using overlap weighting. The propensity score for receiving combined therapy was estimated using male age, female partner age, infertility duration, pathogen category, baseline sperm concentration, and baseline total sperm count. Covariate balance before and after weighting was assessed using standardized mean differences, with an absolute standardized mean difference <0.10 considered indicative of adequate balance. Weighted outcome models were estimated using robust sandwich standard errors. Given the relatively small sample size and the low number of treatment failures for some binary outcomes, the adjusted logistic regression analyses were additionally repeated using Firth penalized likelihood as a sensitivity analysis to reduce small-sample and separation-related bias. The four secondary seminal outcomes were also evaluated using Holm correction for multiple comparisons. For the baseline-adjusted continuous-outcome models, residual and influence diagnostics, functional-form assessment, and multicollinearity were evaluated. Sensitivity analyses additionally examined potentially influential observations and alternative models allowing a non-linear association between the corresponding baseline and post-treatment semen parameter. These additional propensity-score, penalized-regression, multiplicity, and model-diagnostic analyses were considered exploratory sensitivity analyses. All tests were two-sided, and *p* < 0.05 was considered statistically significant. Secondary analyses were considered exploratory.

## 3. Results

**Figure 1:**
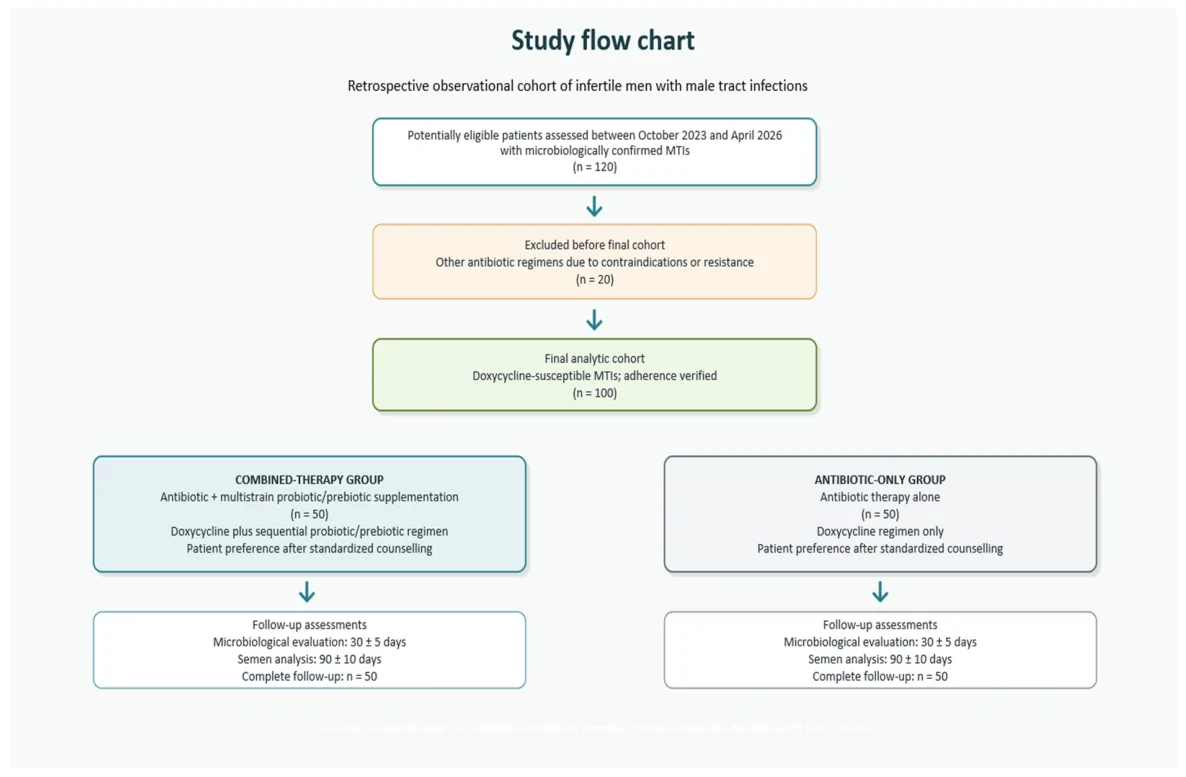
Flow-chart of the study.

Detailed flow-chart of the study is reported in Figure 1.

A total of 100 infertile men with microbiologically confirmed MTIs were included, of whom 50 received antibiotic therapy combined with multistrain probiotic/prebiotic supplementation and 50 received antibiotic therapy alone. No statistically significant differences were detected in the measured baseline characteristics between groups (Table 1). The overall distribution of typical, atypical, and mixed infections did not differ significantly between treatment groups (global *p* = 0.134).

No treatment-related adverse events were documented in the available clinical records.

**Figure 2:**
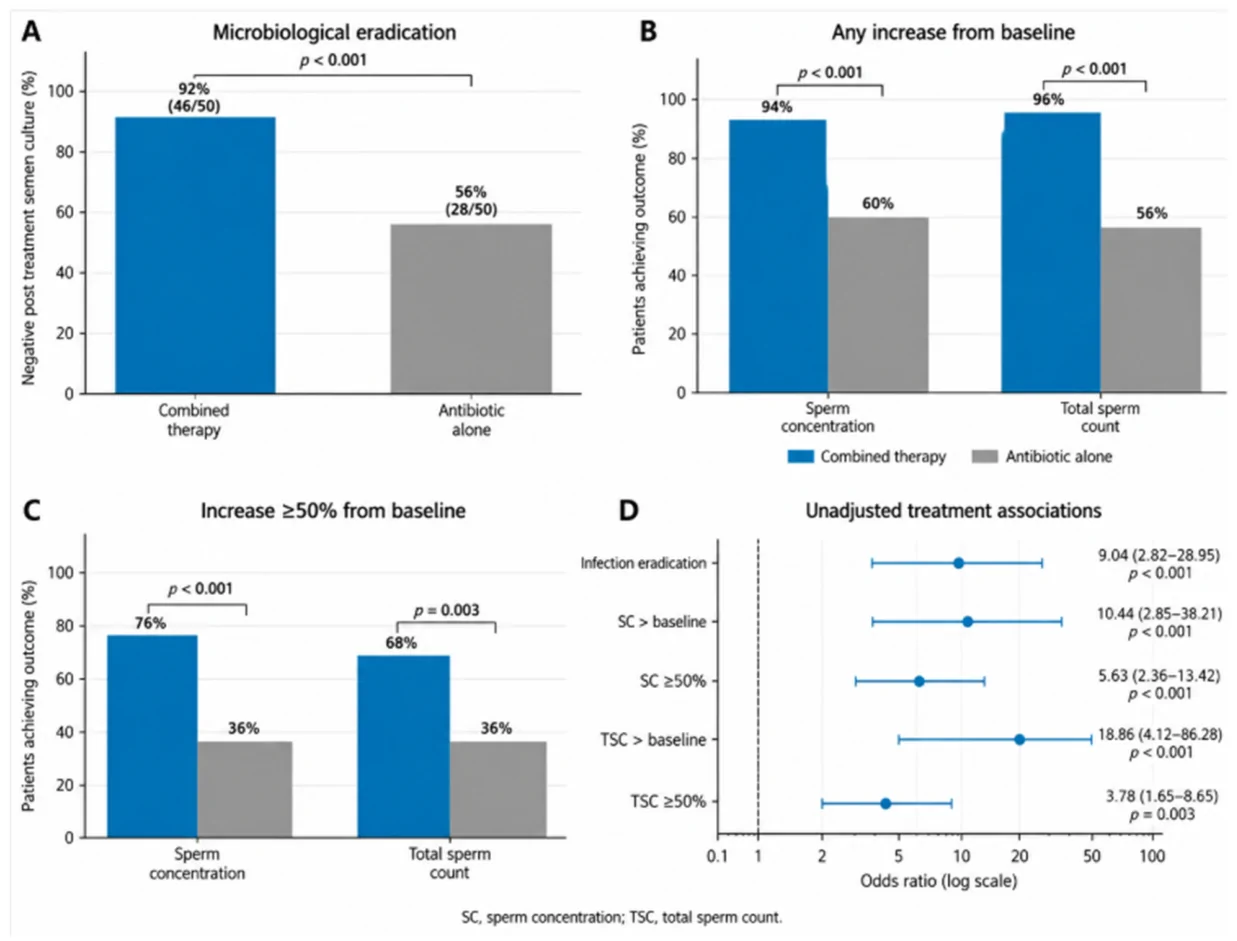
Microbiological and semen outcomes according to treatment regimen. (**A**) Proportion of patients achieving microbiological eradication, defined as a negative post-treatment semen microbiological evaluation. (**B**) Proportion showing any increase from baseline in sperm concentration or total sperm count. (**C**) Proportion achieving an increase of at least 50% over baseline. (**D**) Unadjusted odds ratios and 95% confidence intervals for the association between combined therapy and each outcome. The dashed vertical line indicates the null value (OR = 1). Odds ratios greater than 1 favor combined therapy. Between-group comparisons were performed using Fisher’s exact test. Abbreviations: SC, sperm concentration; TSC, total sperm count; OR, odds ratio.

Microbiological eradication was achieved in 74 of 100 patients (74.0%). A negative post-treatment semen culture was documented in 46 of 50 patients (92.0%) receiving combined therapy and in 28 of 50 patients (56.0%) receiving antibiotic therapy alone, corresponding to an absolute difference of 36.0 percentage points (95% CI 20.3–51.7), an OR of 9.04 (95% CI 2.82–28.95), and *p* < 0.001 (Table 2 and Figure 2).

Any increase in sperm concentration was observed in 47/50 cases (94.0%) and 30/50 controls (60.0%; OR 10.44, 95% CI 2.85–38.21; *p* < 0.001). Using the more stringent criterion of an increase of at least 50% over baseline, response rates were 76.0% and 36.0%, respectively (OR 5.63, 95% CI 2.36–13.42; *p* < 0.001).

Any increase in total sperm count occurred in 48/50 cases (96.0%) compared with 28/50 controls (56.0%; OR 18.86, 95% CI 4.12–86.28; *p* < 0.001), whereas an increase of at least 50% was observed in 34/50 cases (68.0%) and 18/50 controls (36.0%; OR 3.78, 95% CI 1.65–8.65; *p* = 0.003).

In exploratory analyses, no significant between-group difference was observed in the change in total motility (*p* = 0.778) nor progressive motility (*p* = 0.530). Conversely, TMSC increased significantly within the combined-therapy group (*p* < 0.001), but not in the antibiotic-only group (*p* = 0.963). Accordingly, the absolute change in TMSC was significantly greater with combined therapy.

**Figure 3:**
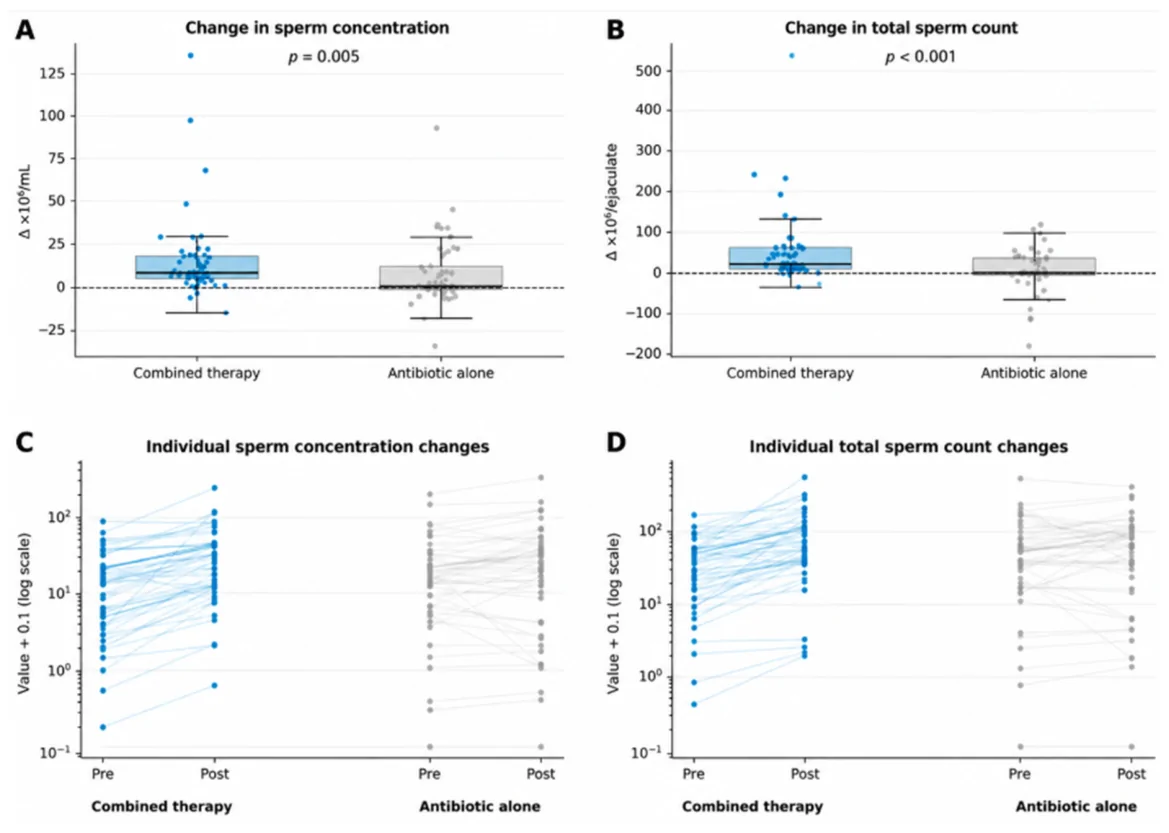
Absolute and individual changes in semen parameters following treatment. (**A**,**B**) Distribution of absolute changes from baseline in sperm concentration and total sperm count, respectively. Boxes represent the interquartile range, horizontal lines indicate the median, whiskers represent values within 1.5 times the interquartile range, and dots represent individual observations. The dashed horizontal line indicates no change from baseline. Between-group comparisons were performed using the Mann–Whitney U test. (**C**,**D**) Individual pre- to post-treatment trajectories for sperm concentration and total sperm count, displayed on a logarithmic scale.

The magnitude of the seminal response was also greater in the combined therapy group when changes were analyzed as continuous variables (Figure 3). The median increase in sperm concentration was 8.60 × 10^6^/mL (IQR 5.12–18.75) in cases and 0.75 × 10^6^/mL (IQR −1.30–12.30) in controls (*p* = 0.005; Cliff’s delta 0.33, 95% CI 0.09–0.55). The corresponding median increase in total sperm count was 23.26 × 10^6^/ejaculate (IQR 11.26–62.50) and 0.55 × 10^6^/ejaculate (IQR −4.64–37.05), respectively (*p* < 0.001; Cliff’s delta 0.43, 95% CI 0.23–0.63). In baseline-adjusted continuous-outcome models, combined therapy remained associated with higher post-treatment values. After adjustment for the corresponding baseline semen parameter, infertility duration, female partner age, and pathogen category, the geometric mean ratio for post-treatment sperm concentration was 1.74 (95% CI 1.35–2.25; *p* < 0.001), while that for total sperm count was 1.96 (95% CI 1.45–2.63; *p* < 0.001).

**Figure 4:**
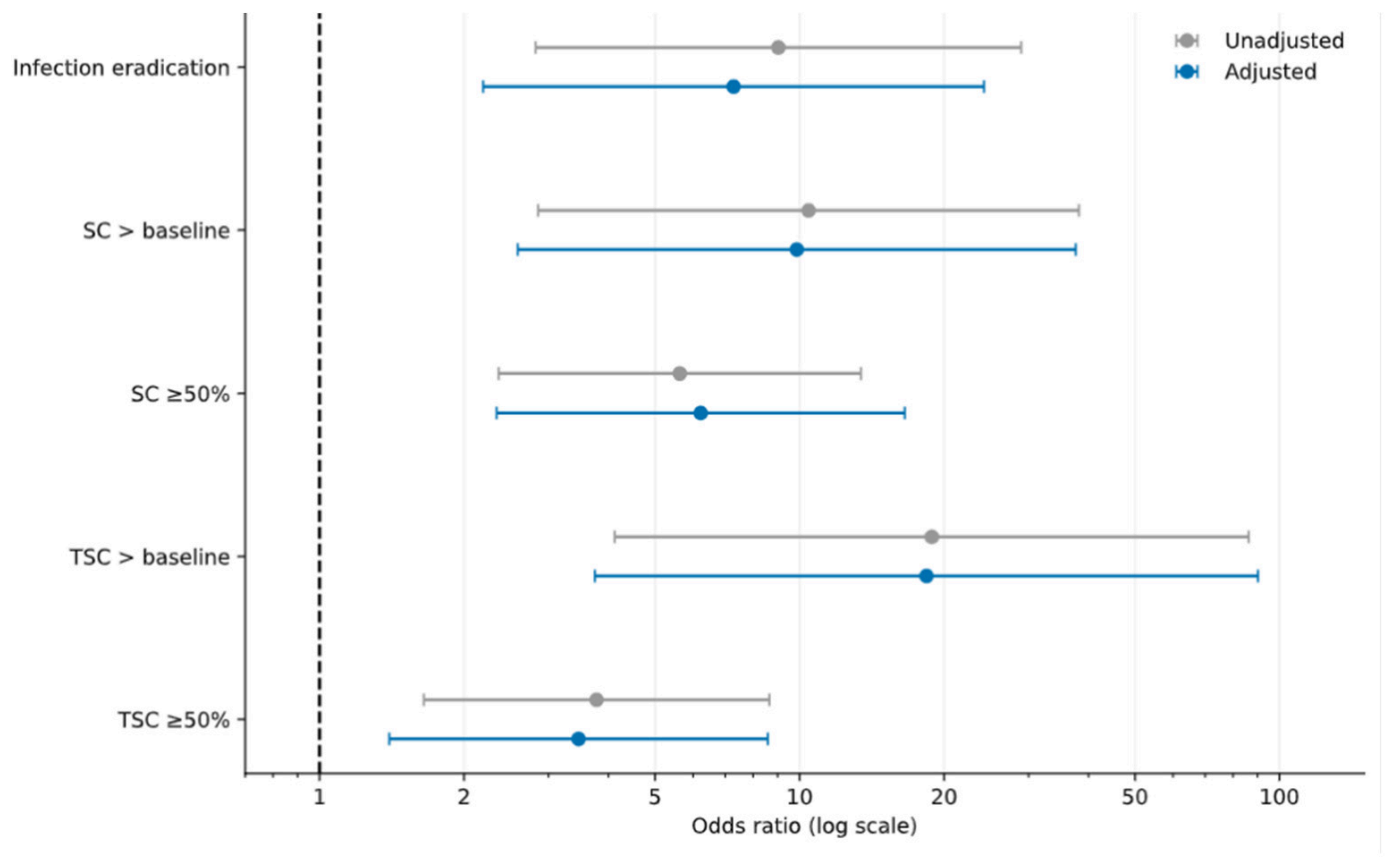
Unadjusted and adjusted associations between combined therapy and treatment outcomes. Points represent odds ratios and horizontal lines represent 95% confidence intervals. The dashed vertical line indicates the null value (OR = 1). Adjusted models included infertility duration, female partner age, and pathogen category; models of seminal outcomes additionally included the corresponding baseline semen parameter. Odds ratios greater than 1 favor combined therapy.

In multivariable logistic regression analyses, all associations remained significant after adjustment for infertility duration, female partner age, pathogen category, and, for seminal outcomes, the corresponding baseline semen parameter. Combined therapy was independently associated with microbiological eradication (adjusted OR 7.29, 95% CI 2.19–24.23; *p* = 0.001), any increase in sperm concentration (adjusted OR 9.87, 95% CI 2.59–37.63; *p* < 0.001), an increase in sperm concentration of at least 50% (adjusted OR 6.23, 95% CI 2.34–16.57; *p* < 0.001), any increase in total sperm count (adjusted OR 18.38, 95% CI 3.75–90.19; *p* < 0.001), and an increase in total sperm count of at least 50% (adjusted OR 3.46, 95% CI 1.40–8.59; *p* = 0.007; Figure 4).

Additional sensitivity analyses supported the robustness of the main findings. In a propensity-score overlap-weighted analysis, balance in the measured baseline characteristics substantially improved after weighting, with all absolute standardized mean differences < 0.10. Combined therapy remained associated with microbiological eradication (overlap-weighted OR 6.58, 95% CI 1.83–23.69; *p* = 0.004), any increase in sperm concentration (OR 8.40, 95% CI 1.89–37.34; *p* = 0.005), a ≥ 50% increase in sperm concentration (OR 5.92, 95% CI 2.26–15.49; *p* < 0.001), any increase in total sperm count (OR 16.25, 95% CI 3.12–84.75; *p* < 0.001), and a ≥50% increase in total sperm count (OR 3.00, 95% CI 1.21–7.44; *p* = 0.018). Similar associations were obtained using Firth penalized logistic regression, with adjusted ORs ranging from 3.22 to 13.90 and all *p* ≤ 0.007. Furthermore, all four secondary seminal responder outcomes remained significant after Holm correction for multiple comparisons (all adjusted *p* ≤ 0.003).

Diagnostic assessment of the baseline-adjusted continuous models showed no relevant multicollinearity or heteroscedasticity and no highly influential individual observation. A sensitivity analysis allowing a non-linear association with the corresponding baseline semen parameter yielded results consistent with the primary continuous models.

The association between combined therapy and microbiological eradication was observed in both major microbiological subgroups. Among patients with typical pathogens, eradication occurred in 19/21 cases (90.5%) and 11/19 controls (57.9%; OR 6.91, 95% CI 1.24–38.52; *p* = 0.028). Among patients with atypical pathogens, eradication occurred in 25/27 cases (92.6%) and 15/23 controls (65.2%; OR 6.67, 95% CI 1.25–35.65; *p* = 0.030). There was no evidence of treatment-effect heterogeneity according to pathogen category (*p* for interaction = 0.977). However, these subgroup analyses were exploratory and the relatively small subgroup sizes and wide confidence intervals limit the ability to assess treatment-effect heterogeneity.

After excluding the 10 patients with mixed infections, combined therapy remained significantly associated with microbiological eradication and with all four definitions of seminal response (all *p* ≤ 0.003).

## 4. Discussion

The present study found that adjunctive multistrain probiotic/prebiotic supplementation was associated with higher short-term microbiological eradication rates and greater improvements in sperm concentration and total sperm count in infertile men with microbiologically confirmed MTIs. These associations remained robust across adjusted analyses, continuous outcome models, pathogen-specific subgroup analyses, and sensitivity analyses, supporting the potential role of microbiota-targeted interventions as an adjunct to conventional antibiotic therapy.

Antibiotic therapy remains the cornerstone of treatment for microbiologically documented MTIs [18]. Nevertheless, microbiological persistence or recurrence after treatment is not uncommon, particularly in patients with chronic or recurrent infections [20]. Several factors may contribute to incomplete eradication, including antimicrobial resistance, inadequate tissue penetration, biofilm formation, persistence of microbial reservoirs, and disruption of the commensal microbiota [38].

Our findings suggest that the multistrain probiotic/prebiotic intervention may be associated with improved eradication through mechanisms that remain to be established. Although the observational design does not allow a causal interpretation, the magnitude and consistency of this association are clinically relevant. Importantly, the effect was observed in both typical and atypical pathogen subgroups, without evidence of treatment-effect heterogeneity according to pathogen category. This finding suggests that the potential benefit of probiotic/prebiotic supplementation may not be restricted to a single microbiological profile, although the relatively small subgroup sizes require cautious interpretation.

The present findings are consistent with the growing recognition that microbial ecosystems may influence reproductive health beyond their role in overt infections [39]. The human microbiota is now considered an important regulator of immune, metabolic and endocrine homeostasis, and alterations in microbial composition have been implicated in chronic inflammatory disorders affecting multiple organs [40], including the male reproductive tract [17].

Over the past decade, the advent of next-generation sequencing has demonstrated that the male genital tract harbors a resident microbiota even in asymptomatic individuals [41], shifting the traditional view that microorganisms detected in semen merely reflect infection. This paradigm shift has stimulated growing interest in the concept of the gut–testis axis, highlighting the potential contribution of the seminal microbiota to male reproductive health and fertility [42,43,44]. Several biological mechanisms have been proposed to explain this relationship, including modulation of local inflammatory responses, oxidative stress, impairment of the blood–testis barrier, and direct effects on sperm function [45].

Alterations in gut microbial composition have been reported in infertile men with MTIs, supporting the hypothesis that intestinal dysbiosis may contribute to chronic inflammation, altered immune regulation, and susceptibility to recurrent infection [17]. Antibiotics may further disrupt microbial homeostasis by reducing potentially beneficial bacterial populations and promoting ecological imbalance [28]. Probiotic and prebiotic supplementation may counteract some of these effects by supporting the restoration of intestinal microbial diversity, improving epithelial barrier function, modulating immune responses, and reducing systemic and local inflammatory signaling [46]. However, the present study did not directly assess intestinal or seminal microbiota composition, inflammatory mediators, oxidative stress, or epithelial permeability. Therefore, these mechanisms remain biologically plausible interpretations rather than demonstrated mediators of the observed clinical associations.

An additional important finding was the greater improvement in sperm concentration and total sperm count among patients receiving combined therapy. MTIs may impair semen quality through several mechanisms, including inflammatory injury, oxidative stress, altered accessory gland secretion, direct microbial effects on spermatozoa, and partial obstruction of the seminal tract [16,47]. The higher microbiological eradication rate in the combined therapy group may therefore have contributed to a more favorable environment for spermatogenesis and sperm transport.

An important point concerns the differential response of sperm concentration and motility. While combined therapy was associated with greater improvements in sperm concentration and total sperm count, no significant between-group differences were observed in total or progressive motility. This finding may reflect the fact that sperm output and sperm functional maturation are influenced by partially distinct biological processes. In particular, infection- and inflammation-related oxidative stress may exert a persistent detrimental effect on sperm membrane integrity, mitochondrial function, and flagellar activity, which may not be rapidly reversed immediately following microbiological eradication. Thus, clearance of the infectious stimulus may be more readily reflected in terms of resolution of sub-obstruction and sperm output. However, these mechanistic interpretations remain speculative, as oxidative stress, mitochondrial function, and other molecular determinants of sperm motility were not directly assessed in the present study.

Combined therapy was associated with larger absolute increases in both sperm concentration and total sperm count, and the association persisted in baseline-adjusted continuous-outcome models. This consistency across binary and continuous analyses strengthens the internal coherence of the findings.

Nevertheless, any increase above baseline is a sensitive but potentially unstable definition because semen parameters show considerable intraindividual biological variability. The more stringent threshold of a ≥50% increase and the baseline-adjusted continuous analyses are therefore particularly important, as they provide additional support for a genuine treatment-associated difference rather than a minimal fluctuation around baseline.

Previous studies evaluating probiotic supplementation in male infertility have primarily focused on semen parameters [48]. By contrast, our study specifically focused on infertile men with microbiologically confirmed MTIs and demonstrated an association between microbiota-targeted therapy and both pathogen eradication and recovery of semen quality. These findings therefore extend the current evidence beyond symptomatic improvement, suggesting a potential role for adjunctive microbiota modulation in enhancing the effectiveness of conventional antimicrobial treatment in infertile patients.

The clinical implications of this hypothesis-generating study should, however, be interpreted cautiously. This study suggests that adjunctive supplementation could have meaningful clinical utility, particularly in patients at risk of persistent infection or incomplete recovery of semen quality. However, probiotic/prebiotic supplementation cannot yet be considered a standard component of treatment for MTIs. The current evidence remains limited, and the optimal strains, doses, duration, timing, and patient selection criteria have not been established. In addition, because only one post-treatment microbiological assessment was available, the present study should be interpreted as assessing short-term microbiological eradication rather than confirmed microbiological cure or sustained prevention of recurrence or reinfection.

Furthermore, improvement in semen parameters does not necessarily translate into higher rates of natural conception, pregnancy, or live birth [1]. Future studies should therefore include reproductive outcomes and longer follow-up.

Although the present findings should be considered hypothesis-generating, they provide a rationale for testing microbiota-targeted adjunctive strategies in prospective randomized clinical trials. Several limitations must also be acknowledged. First, the retrospective and non-randomized design introduces the possibility of selection bias and residual confounding. Treatment allocation was based on patient preference, and patients choosing adjunctive supplementation may have differed from those choosing antibiotic therapy alone in unmeasured characteristics, including motivation, health-related behaviors, adherence, or other lifestyle factors that could have influenced treatment response. Although only patients with verified compliance and adherence were included, differences in adherence beyond the predefined treatment period or in other health-related behaviors cannot be excluded.

Residual confounding cannot be completely excluded despite multivariable adjustment. The limited sample size reduced the precision of some estimates, as reflected by the wide confidence intervals, particularly for subgroup and high-response analyses. Second, the study was conducted at a single center and included a relatively limited sample size, resulting in wide confidence intervals for some estimates. Third, microbiota composition was not directly assessed, preventing confirmation that supplementation effectively modified the intestinal or seminal microbial ecosystem. Fourth, inflammatory markers, oxidative stress parameters, sperm DNA fragmentation, and reproductive outcomes were not evaluated. In addition, semen analysis is subject to substantial biological variability, and the use of a single pre- and post-treatment assessment may not fully capture each patient’s underlying seminal status. Moreover, the adjunctive regimen was a multicomponent intervention including several probiotic strains and prebiotic substrates administered sequentially. Consequently, the present study cannot determine whether the observed associations were attributable to a specific strain, prebiotic component, treatment phase, or interaction among components. Finally, reproductive outcomes, including natural pregnancy and assisted reproductive technology outcomes, were not systematically available and therefore could not be evaluated. Consequently, whether the observed microbiological and seminal associations translate into improved reproductive outcomes remains unknown.

Despite these limitations, several methodological aspects strengthen the internal validity of our findings. Patients underwent standardized diagnostic work-up, identical antibiotic therapy, centralized semen evaluation, predefined follow-up, and multiple complementary statistical analyses. The inclusion of an antibiotic-only comparator allowed the potential additional contribution of probiotic/prebiotic supplementation to be evaluated. The findings were supported by multiple analytical approaches, including responder analyses, continuous outcomes, multivariable models, pathogen-specific analyses, and sensitivity analysis excluding mixed infections.

## 5. Conclusions

Adjunctive multistrain probiotic/prebiotic supplementation was associated with higher short-term microbiological eradication rates and greater improvements in sperm concentration and total sperm count compared with antibiotic therapy alone in this retrospective cohort of infertile men with clinically relevant MTIs. These hypothesis-generating findings remained consistent across several adjusted and sensitivity analyses but cannot establish causality because treatment allocation was non-randomized. Adequately powered randomized controlled trials are required before any clinical recommendation regarding routine adjunctive probiotic/prebiotic supplementation can be made.

## Figures and Tables

**Table 1:** Baseline characteristics according to treatment group. Abbreviations: FSH: follicle-stimulating hormone; LH: luteinizing hormone.

Variable	Normal Values	Overall (n = 100)	Cases (n = 50)	Controls (n = 50)	*p*-Value
Age (years)	n.a.	36.1 ± 7.8	36.2 ± 8.7	36.0 ± 6.7	0.889
Female partner age (years)	n.a.	33.5 ± 5.7	34.1 ± 5.8	33.0 ± 5.6	0.363
Infertility duration (years)	n.a.	2.0 (1.0–2.1)	2.0 (1.0–2.0)	2.0 (1.0–2.9)	0.310
FSH (mIU/L)	1.5–8.0	4.8 (3.5–6.0)	4.7 (3.5–6.1)	4.8 (3.5–5.8)	1.000
LH (mIU/L)	1.5–9.4	4.0 (2.9–6.0)	4.0 (2.7–5.8)	4.2 (3.0–6.0)	0.736
Total testosterone (nmol/L)	>12.0	15.8 (12.7–19.6)	15.6 (12.8–20.0)	15.9 (12.5–18.0)	0.799
Mean testicular volume (mL)	>11.5	14.0 (12.2–16.0)	14.0 (11.7–16.0)	14.0 (12.9–16.0)	0.256
Distribution of pathogens					0.134
Typical pathogens, n (%)	n.a.	40 (40.0)	21 (42.0)	19 (38.0)	
Atypical pathogens, n (%)	n.a.	50 (50.0)	27 (54.0)	23 (46.0)	
Combined typical and atypical pathogens, n (%)	n.a.	10 (10.0)	2 (4.0)	8 (16.0)	
Sperm concentration (×10^6^/mL)	>16.0	14.8 (5.0–22.7)	13.9 (4.3–21.0)	16.5 (6.4–24.8)	0.422
Total sperm count (×10^6^/ejaculate)	>39.0	39.8 (16.0–66.1)	37.8 (16.0–59.5)	48.5 (16.9–83.6)	0.226
Total motility (%)	>42.0	40.0 (25.0–59.0)	39.5 (22.5–61.5)	45.0 (30.0–58.0)	0.695
Progressive motility (%)	>30.0	25.5 (15.3–41.0)	27.0 (14.0–43.5)	25.0 (17.0–38.5)	0.909
Total motile sperm count (×10^6^)	n.a.	16.0 (5.3–38.7)	9.9 (4.6–25.0)	19.2 (6.3–46.3)	0.203

**Table 2:** Microbiological eradication and seminal response according to treatment group. Data are presented as n/N (%). Risk differences are expressed in percentage points and compare combined therapy with antibiotic therapy alone. Odds ratios greater than 1 favor combined therapy. Between-group comparisons were performed using Fisher’s exact test. CI, confidence interval; OR, odds ratio.

Outcome	Cases, n/N (%)	Controls, n/N (%)	Risk Difference, % (95% CI)	OR (95% CI)	*p*-Value
Microbiological eradication	46/50 (92.0)	28/50 (56.0)	36.0 (20.3 to 51.7)	9.04 (2.82–28.95)	<0.001
Sperm concentration > baseline	47/50 (94.0)	30/50 (60.0)	34.0 (18.9 to 49.1)	10.44 (2.85–38.21)	<0.001
Sperm concentration ≥ 1.5-fold baseline	38/50 (76.0)	18/50 (36.0)	40.0 (22.2 to 57.8)	5.63 (2.36–13.42)	<0.001
Total sperm count > baseline	48/50 (96.0)	28/50 (56.0)	40.0 (25.2 to 54.8)	18.86 (4.12–86.28)	<0.001
Total sperm count ≥ 1.5-fold baseline	34/50 (68.0)	18/50 (36.0)	32.0 (13.4 to 50.6)	3.78 (1.65–8.65)	0.003

## Data Availability

No new data were created or analyzed in this study. Data sharing is not applicable to this article.
